# MicroRNA-138-1-3p sensitizes sorafenib to hepatocellular carcinoma by targeting PAK5 mediated β-catenin/ABCB1 signaling pathway

**DOI:** 10.1186/s12929-021-00752-4

**Published:** 2021-08-02

**Authors:** Tong-tong Li, Jie Mou, Yao-jie Pan, Fu-chun Huo, Wen-qi Du, Jia Liang, Yang Wang, Lan-sheng Zhang, Dong-sheng Pei

**Affiliations:** 1 Department of Pathology, Xuzhou Medical University, 209 Tongshan Road, Xuzhou, Jiangsu 221004 People’s Republic of China; 2 Jiangsu Key Laboratory of New Drug and Clinical Pharmacy, School of Pharmacy, Xuzhou Medical University, 209 Tongshan Road, Xuzhou, 221006 China; 3 Department of Oncological Radiotherapy, The Second Affiliated Hospital of Xuzhou Medical University, Xuzhou, China; 4 Department of Pathology and Pathophysiology, Jiangsu Vocational College of Medicine, Yancheng, 224005 Jiangsu China

**Keywords:** Hepatocellular carcinoma, Sorafenib, MiR-138-1-3p, p21-Activated kinases 5, Wnt/β-catenin pathway, ABCB1

## Abstract

**Background:**

Sorafenib is a kinase inhibitor that is used as a first-line therapy in advanced hepatocellular carcinoma (HCC) patients. However, the existence of sorafenib resistance has limited its therapeutic effect. Through RNA sequencing, we demonstrated that miR-138-1-3p was downregulated in sorafenib resistant HCC cell lines. This study aimed to investigate the role of miR-138-1-3p in sorafenib resistance of HCC.

**Methods:**

In this study, quantitative real-time PCR (qPCR) and Western Blot were utilized to detect the levels of PAK5 in sorafenib-resistant HCC cells and parental cells. The biological functions of miR-138-1-3p and PAK5 in sorafenib-resistant cells and their parental cells were explored by cell viability assays and flow cytometric analyses. The mechanisms for the involvement of PAK5 were examined via co-immunoprecipitation (co-IP), immunofluorescence, dual luciferase reporter assay and chromatin immunoprecipitation (ChIP). The effects of miR-138-1-3p and PAK5 on HCC sorafenib resistant characteristics were investigated by a xenotransplantation model.

**Results:**

We detected significant down-regulation of miR-138-1-3p and up-regulation of PAK5 in sorafenib-resistance HCC cell lines. Mechanistic studies revealed that miR-138-1-3p reduced the protein expression of PAK5 by directly targeting the 3′-UTR of PAK5 mRNA. In addition, we verified that PAK5 enhanced the phosphorylation and nuclear translocation of β-catenin that increased the transcriptional activity of a multidrug resistance protein ABCB1.

**Conclusions:**

PAK5 contributed to the sorafenib resistant characteristics of HCC via β-catenin/ABCB1 signaling pathway. Our findings identified the correlation between miR-138-1-3p and PAK5 and the molecular mechanisms of PAK5-mediated sorafenib resistance in HCC, which provided a potential therapeutic target in advanced HCC patients.

## Background

Hepatocellular carcinoma (HCC) has a high fatality rate and patients are always diagnosed at advanced stages [1]. As a result, systemic treatment plan with an emphasis on targeted therapy is considered as a better option. Sorafenib is the Food and Drug Administration (FDA)-approved first-line targeted drug for advantage HCC patients [2, 3]. Sorafenib inhibits tumor growth by targeting angiogenesis and tumorigenesis pathways [4]. However, sorafenib is incapable of eradicating the neoplasm [5]. Only approximately 30% of patients can benefit from sorafenib due to acquired drug resistance [6]. Thus, understanding the resistance mechanism(s) of sorafenib in HCC has pivotal significance.

Previous studies have shown that epidermal growth factor receptor (EGFR), AKT and MAPK activation may inhibit sorafenib-induced apoptosis in HCC cells [7–9]. Hypoxic environment was essential microenvironmental characteristics of sorafenib resistance cells. Hypoxia-inducible factor (HIF)-1α and HIF-2α, as important components, could regulate the adaptation of hypoxia in cancer cells and are high-expressed in sorafenib-resistant HCC specimens [10, 11]. In addition, epithelial-mesenchymal transition (EMT) was a principal feature of sorafenib resistance. In sorafenib-resistant cells, E-Cadherin (epithelial marker) was decreased while N-Cadherin, Vimentin, and Snail (mesenchymal markers) were increased [12, 13]. Meanwhile, Karyopherin subunit alpha 3 (KPNA3) was found to trigger EMT and mediate drug resistance [14]. Similarly, cancer stem cells (CSCs) or tumor-initiating cells (TICs) was also an important metabolic characteristic that contributed to sorafenib resistance [15, 16].

MicroRNAs (miRNAs) are putative candidate to dampen gene expressions and curb their impact at the post-transcriptional level through binding to the 3'-untranslated region (3′-UTR) of mRNAs [17, 18]. In recent studies, several miRNAs have been indicated as a critical molecule in the development of acquired sorafenib resistance [19, 20]. However, the biological role of miRNAs in HCC sorafenib resistance continues to be ambiguity. In our study, we investigated the effects of miR-138-1-3p in HCC sorafenib-resistant cell lines.

p21-Activated kinases 5 (PAK5) is a conserved serine/threonine protein kinase, which is a modulator of cytoskeleton variations, anti-apoptosis and proliferation in cells [21]. Previous studies from our lab and others’ have shown that PAK5 expression augmented in the tumor cells and promoted resistance of antineoplastic drugs [22–25]. In the current study, PAK5 overexpression was detected in HCC sorafenib-resistant cells.

As an evolutionarily conserved signaling pathway, Wnt/β-catenin signaling pathway not only participates in embryogenesis, but also in cancer cell survival, invasion, migration and multidrug-chemoresistance [26, 27]. P-glycoprotein is an ATP-binding Cassette (ABC) efflux transporter distributing on the cell membrane extensively. Functionally, p-glycoprotein shares a protective mechanism that promote tumor cells survival by the efflux of intracellular drug [28]. In this study, we found that PAK5 elevated the phosphorylation and nuclear translocation of β-catenin. Then, the nucleus p-β-catenin regulated the multidrug resistant protein ABCB1 transcriptional activation.

## Methods

### Cell culture

The human HCC cell lines HepG2 and Hep3B were purchased from the Cell Bank: China Academy of Sciences (Shanghai, China). HepG2, Hep3B and HEK293T cells were cultured in Dulbecco’s modified Eagle’s medium (Hyclone) supplemented with 10% (v/v) FBS. All cells were cultivated at 37 °C with 5% CO_2_.

### Generation of drug-resistant cells

Hep3B and HepG2 were cultured with sorafenib at the concentration of 10 μM for 48 h. Viable cells remaining continue to be treated with sorafenib with increasing concentration. Cell lines were considered to be sorafenib resistant when the resistant drug index examined up to three.

### Transfection and stable cell line generation

The miRNAs mimics, mimics-NC, miR-138-1-3p inhibitor, inhibitor-NC and PAK5 siRNA (si-PAK5, 5′-CAAAGTCTTCGTACCTGAATC-3′) were obtained from Gene Pharma (Shanghai, China) and were transfected in Hep3B and HepG2 cells using siLentFect Lipid Reagent (Bio-Rad, Hercules, CA, USA). The PAK5/Vector and Myc-PAK5 expression plasmids were purchased from Guangzhou FulenGen Co. Hep3B and HepG2 cells were transiently transfected with PAK5 plasmids using X-tremeGENE HP DNA Transfection Reagent (Roche, Indianapolis, IN, USA). All transfection procedures follow the manufacturer’s instructions. The LV-miR-138-1-3p-NC and LV-miR-138-1-3p (miR-138-1-3p, 5’-GCUACUUCACAACACCAGGGCC-3’) were established by Gene Pharma (Shanghai, China). HepG2 were transfected with lentivirus for 48 h, and then selected via puromycin (Vicmed, China) for 30 days.

### Cell viability assay

Cell viability was assayed by the Cell Counting Kit-8 (CCK-8) kit (Beyotime, China). Post-treatment Hep3B and HepG2 cells (2 × 10^3^) were cultured in 96-well microplate (Corning Incorporated, New York, USA) in quintuplicate at 37 °C with 5% CO_2_. Then, CCK-8 reagent (10 μl) and 90 μl serum-free medium were added to each well at 24, 48, 72, and 96 h, respectively. Absorbance was measured at 450 nm after 1 h incubation at 37 °C by a multi-function enzyme-linked analyzer (Biotek Instruments, Winooski, VT, USA).

### RNA isolation, reverse transcription and quantitative real-time PCR (qRT-PCR)

Total RNA isolation was performed using Trizol Reagent (Takara, Dalian, China) following the manufacturers’ instructions. RNA reverse transcription was performed with the PrimeScript™ RT reagent Kit (Perfect Real Time) (Takara, Dalian, China) and program set according to the operating instructions. MiRNA levels were determined by BrightGreen Express 2X qPCR MasterMix (Abm, Canada) with a 7500 Real-time PCR System (Life Technologies, NY, USA). Relative quantitation of miRNAs was normalized to U6 levels, PAK5, ABCB1, ABCG2, LRP and MRP2 were normalized to GAPDH levels (Sangon, Shanghai, China). Relative RNA expression was calculated via the comparative threshold cycle (Ct) method (relative gene expression = 2– (ΔCtsample − ΔCtcontrol)). The primers used are described in Table 1.

**Table 1:** Primer sequences

	Forward primer	Reverse primer
miR-138-1-3p	5′-CGCGGCTACTTCACAACACC-3′	5′-AGTGCAGGGTCCGAGGTATT-3′
U6	5′-GCTTCGGCAGCACATATACTAAAAT-3′	5′-CGCTTCACGAATT TGCGTGTCAT-3′
PAK5	5'-GGCGTCCTCTTGTGTCTTC-3'	5'-GTACTGAGTCCTTCTGATTTGC-3'
β-catenin	5'-GGGTCCTCTGTGAACTTGCTC -3'	5'-TTCTTGTAATCTTGTGGCTTGTCC -3'
ABCB1	5′-GGCCTAATGCCGAACACATT-3′	5′-CAGCGTCTGGCCCTTCTTC-3′
ABCG2	5′-CAGGTGGAGGCAAATCTTCGT-3′	5′-ACACACCACGGATAAACTGA-3′
LRP	5′-GTCTTCGGGCCTGAGCTGGTGTCG-3′	5′-CTTGGCCGTCTCTTGGGGGTCCTT-3′
MRP2	5′-CCAAAGACAACAGCTGAAA-3′	5′-TACTTGGTGGCACATAAAC-3′
GAPDH	5′-TGGTATCGTGGAAGGACTCAT-3′	5′-ATGCCAGTGAGCTTCCCGTTCAGC-3′

### Flow cytometric analysis

Post-treatment Hep3B and HepG2 cells were resuspended in PBS. Annexin V-FITC Apoptosis Detection Kit (Beyotime, China) and flow cytometry (Becton Dickinson, Franklin Lakes, NJ, USA) were visualized to evaluate the percentage of apoptotic cells. Data were analyzed using a FACSCalibur Flow Cytometer (Becton Dickinson).

### Antibodies and Western Blot

Cell lysis and total protein extraction were performed using the RIPA lysis buffer (KeyGen BioTECH, Jiangsu, China) and protein concentrations were gauged by Enhanced BCA Protein Assay Kit (KeyGen BioTECH). Nuclear and cytoplasmic protein was distilled via Nuclear and Cytoplasmic Protein Extraction Kit (KeyGen BioTECH). SDS-PAGE electrophoresis and nitrocellulose blotting membranes (thermo fisher scientific) were used for proteins transfer. The specific primary antibodies were incubated overnight at 4 °C, including anti-PAK5 (1:1000, Abcam, Shanghai, China), anti-ABCB1 (1:500, Abcam, Shanghai, China), anti-β-catenin (1:1000, Santa Cruz, USA), anti-p-β-catenin (S675) (1:1000, Cell Signaling Technology, USA) and β-actin (1:5000, proteintech, China). Anti-rabbit HRP or Anti-Mouse HRP (1:10,000, Vicmed) were incubated at room temperature for 2 h afterwards. Western Blot images were detected via Chemistar™ High-sig ECL Western Blot Substrate (Tanon, shanghai, China).

### Rhodamine 123 efflux assay

Rhodamine 123 thoroughly dissolved in DMSO at a concentration of 5 mol/L. Seed post-treatment cells on Glass Bottom Culture Dishes (NEST, Wuxi, China) one day in advance. 5 μM Rho123 were added to the medium and incubated for 30 min at 37 °C with 5%CO_2_. Transpose new complete medium. Images were observed by immunofluorescence confocal laser scanning microscopy (Zeiss LSM 880).

### Immunofluorescence

Seed post-treatment cells on Glass Bottom Culture Dishes (NEST, Wuxi, China) one day in advance. Cells were fixed with 4% paraformaldehyde for 20 min, and then blocked with TBS (0.3% Triton X-100 and 0.25% BSA) at room temperature for 2 h. Afterwards, they were incubated overnight at 4 °C with primary antibodies: anti-PAK5 (1:100, Abcam, Shanghai, China) and β-catenin (1:100, Santa Cruz, USA) Afterwards, they were washed three-fold with PBD. Afterwards they were stained with fluorescent secondary antibodies: CoraLite488–conjugated Affinipure Goat Anti-Mouse IgG(H + L) and CoraLite594–conjugated Goat Anti-Rabbit IgG(H + L) (1:200, proteintech, China) at room temperature for 60 min. Nuclei were deal with 4′, 6-Diamidino-2-phenylindole (DAPI) (KeyGen BioTECH) for 10 min. Pictures were taken by immunofluorescence confocal laser scanning microscopy (Zeiss LSM 880).

### Co-immunoprecipitation (co-IP)

Cells were lysed in IP lysed buffer (KeyGen BioTECH) with cocktail of protease/phosphatase inhibitors (1:100, Sigma Aldrich, MO, USA), and then added anti-PAK5 (1:100, Santa Cruz, USA) and Mouse IgG (Beyotime) incubation overnight at 4 °C. 40 μl Protein A/G beads (Santa Cruz) were used for binding to antibodies. Wash beads and heat with 2 × loading buffer. Immunoprecipitated proteins were examined by Western Blot.

### Chromatin immunoprecipitation (ChIP)

ChIP assay kit (Cell Signaling Technology) was used for ChIP assay. 1 × 10^7^ cells were prepared for the first-step. Then DNA complexes were immunoprecipitated with β-catenin antibody. The resulting precipitated DNA samples were quantified by real-time PCR.

### Luciferase reporter assay

The luciferase PAK5/Vector pGL3-Basic and pGL3-ABCB1 were co-transfected into Hep3B and HepG2 cells that lysis in the 48-well plates by using X-tremeGENE HP DNA Transfection Reagent (Roche, Indianapolis, IN, USA). Renilla luciferase activate was used as normalization. Relative luciferase activity was detected 48 h after transfection following manufacturer’s instructions (Promega, USA) using an Orion Microplate Luminometer (Berthold Detection System).

### Clinical specimens

40 Pairs of HCC tumor tissues and corresponding adjacent normal tissues were collected from patients with HCC who underwent surgery at Affiliated Hospital of Xuzhou Medical University (Xuzhou, China). Patients did not receive any radiotherapy or chemotherapy before surgery. The study protocol was approved by the Research Ethics Committee of Xuzhou Medical University. Clinical specimens were obtained with the informed consent of patients.

### Xenograft transplantation model

The 4–6 weeks BALB/c female nude mice were customized by HFK Bioscience (Beijing, China) and randomized into four groups (n = 10 for each): (1) hypodermic inject 2 × 10^6^ LV-ctrl-HepG2-SR cells, (2) hypodermic inject 2 × 10^6^ LV-ctrl-HepG2-SR cells and oral sorafenib 30 mg/kg, twice a day, (3) hypodermic inject 2 × 10^6^ LV-mimics-HepG2-SR cells and oral sorafenib 30 mg/kg, twice a day, (4) hypodermic inject 2 × 10^6^ LV-siPAK5-mimics-HepG2-SR and oral sorafenib 30 mg/kg, twice a day. Two months thereafter, mice were killed with the neoplasms for immunohistochemical staining. All animal experiments were in conformance with the ARRIVE (Animal Research: Reporting of In Vivo Experiments) guidelines and in accordance with the National Institutes of Health Guide for the Care and Use of Laboratory Animals.

### Statistical analysis

Statistical analysis was performed by SPSS 21.0 software (SPSS, USA), and images were acquired with GraphPad Prism 5 software (La Jolla, USA). The significance of the differences between the groups was evaluated by paired two-tailed Student’s t-test or one-way analysis of variance (ANOVA). All results that represented were from at least three independent experiments. Quantitative RT-PCR, luciferase reporter and cell proliferation assays were performed with triplicate duplications. Data represent the mean ± standard deviation (SD). Differences were considered statistically significant when *P* < 0.05 (*P < 0.05, **P < 0.01, ***P < 0.001).

## Results

### miR-138-1-3p expression was reduced in sorafenib-resistant cells and may participate in resistance development

**Fig. 1:**
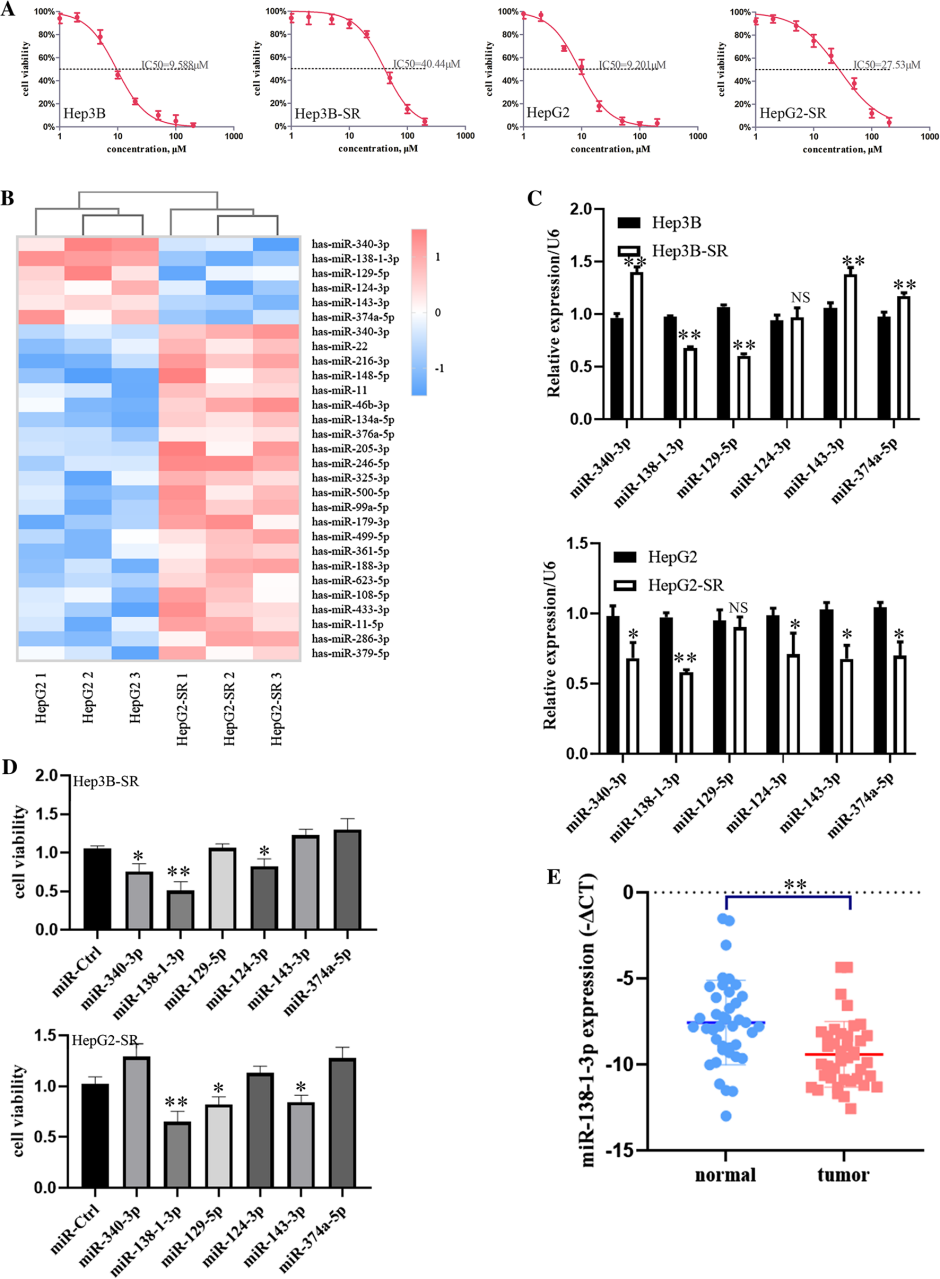
miR-138-1-3p expression was reduced in sorafenib-resistant cells and may participate in resistance development. **A** 50% inhibitory concentration (IC50) measured by CCK-8 assay at different concentration of sorafenib over 48 h. **B** Twenty-nine miRNAs exhibited significantly different expression between the two groups by Heat map from RNA sequencing. six candidates: miR-340-3p, miR-138-1-3p, miR-129-5p, miR-124-3p, miR-143-3p and miR-374a-5p were reduced in HepG2-SR cells; **C** Expression of the six candidates in the resistant cell lines detected by qRT-PCR. **D** Cell viability measured by CCK-8 assay of the resistant cell lines after transfected with six nominated miRNAs mimics, treating with sorafenib (10 μM, 48 h). miR-138-1-3p consistently suppressed resistant cell proliferation. **E** The expression of miR-138-1-3p in tumor tissues and adjacent normal tissues from 40 HCC patients was tested by qRT-PCR

To explore the underlying mechanisms of sorafenib resistance in HCC, we introduced the sorafenib-resistant cell models via chronic exposure to sorafenib in HCC cell lines. We established two sorafenib-resistant cell lines, Hep3B-SR and HepG2-SR. Cell viability assay was utilized to detected the 50% inhibitory concentration (IC50) of sorafenib in Hep3B-SR, HepG2-SR and their parental counterparts. Resistant cell lines were always with a higher IC50 value in the presence of sorafenib than the parental cell lines (Fig. 1A). Evidences showed that miRNAs own great potentials in combination cancer therapy [29–31]. For example, targeting miR-21 could enhance conventional chemotherapeutic efficacy, together with overcoming drug resistance and cancer recurrence [32]. To examine the potential involvement of miRNAs in our study, we performed RNA sequencing on HepG2-SR cells and its parental cells, to identify potential miRNA candidates involved in the process of resistance development. Twenty-nine miRNAs were different expressed between the two groups, and six miRNAs (miR-340-3p, miR-138-1-3p, miR-129-5p, miR-124-3p, miR-143-3p and miR-374a-5p) were down-regulated in HepG2-SR cells by RNA sequencing (Fig. 1B). Then, qRT-PCR analyzed the expression of the six candidates in the resistant cells and its parental cell lines. Significantly low expression of miR-138-1-3p was detected in the sorafenib-resistant HCC cell lines (Fig. 1C). To confirm the biological functions of these miRNAs, the resistant cell lines were transfected with six nominated miRNAs mimics, and cell viability assay analyzed their responses to sorafenib respectively (20 μM, 48 h). miR-138-1-3p mimics consistently sensitized the resistant cell lines to sorafenib (Fig. 1D). We further tested the expression of miR-138-1-3p in tumor tissues and adjacent normal tissues from 40 HCC patients by qRT-PCR, and significant decrease of miR-138-1-3p was found in tumor tissue (Fig. 1E), indicating that miR-138-1-3p is important in HCC development.

### miR-138-1-3p sensitized HCC cells to sorafenib

**Fig. 2:**
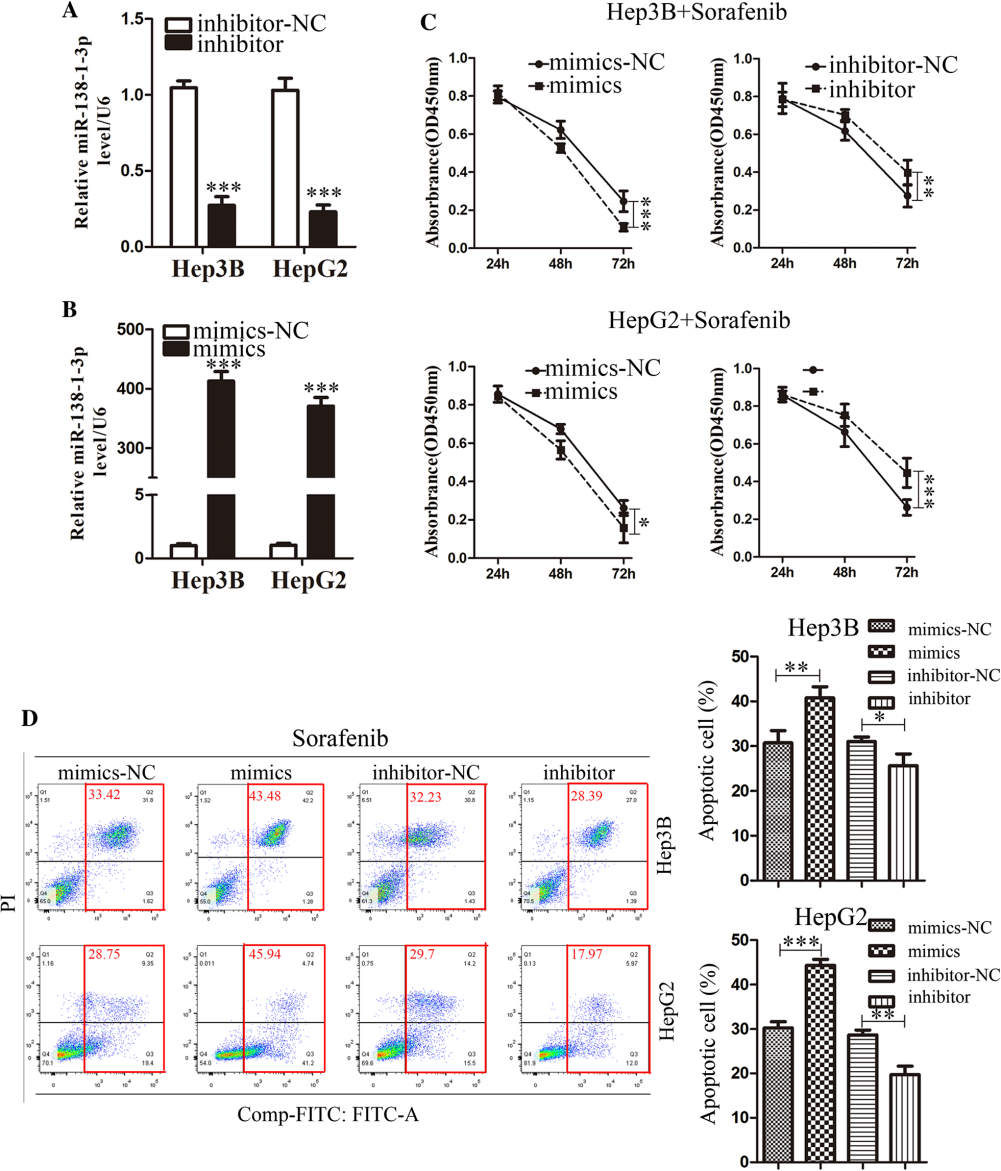
miR-138-1-3p sensitized HCC cells to sorafenib. **A** and **B** qRT-PCR was conducted to determine the transfection efficiency of miR-138-1-3p inhibitor and mimics in Hep3B and HepG2 cells. **C** and **D** The cell viability and flow cytometric analysis were used to assess the influence of miR-138-1-3p overexpression or knockdown on cell viability and apoptosis in Hep3B and HepG2 cells

To investigate the effects of miR-138-1-3p in attained resistance of sorafenib in HCC cells, transient transfection was performed for gain and loss of function studies by miR-138-1-3p mimics and miR-138-1-3p inhibitor, respectively. The transient transfection effectiveness of miR-138-1-3p (mimics and inhibitor) in Hep3B and HepG2 cells was examined via qRT-PCR (Fig. 2A, B). 10 μM was a specific concentration that we chose to get significant difference between the transfected Hep3B and HepG2 cells with control group. Thus, the cell viability assay was tested in indicated sorafenib concentrations (10 μM) at 24 h, 48 h and 72 h, demonstrating that miR-138-1-3p sensitized Hep3B and HepG2 cells to sorafenib treatment. Accordingly, miR-138-1-3p inhibitor significantly promoted sorafenib resistance in Hep3B and HepG2 cells (Fig. 2C). Flow cytometric analysis showed that transfection miR-138-1-3p mimics in Hep3B and HepG2 cells promoted the sorafenib-mediated apoptosis while miR-138-1-3p inhibitor attenuated the cell cytotoxicity of sorafenib (Fig. 2D). Our hypothesis was preliminarily verified.

### miR-138-1-3p involved in sorafenib resistance by targeting PAK5

**Fig. 3:**
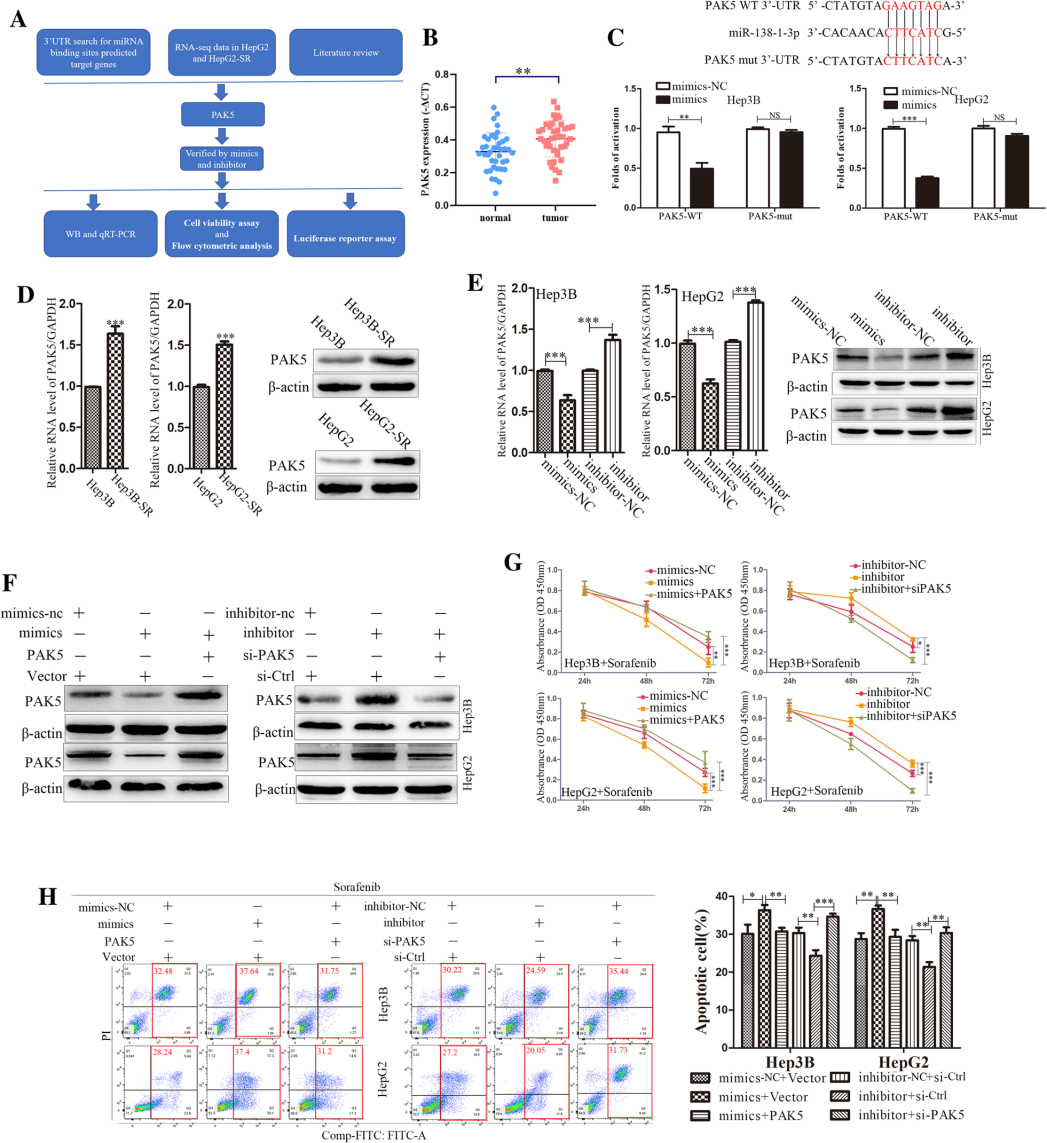
miR-138-1-3p involved in sorafenib resistance by targeting PAK5. **A** The process of research for target genes. **B** The expression of PAK5 in tumor tissues and adjacent normal tissues from 40 HCC patients was tested by qRT-PCR. **C** The predicted binding sequences between miR-138-1-3p and PAK5 by web-based softwares (TargetScan and miRTarBase). The red section was a sign of mutated bases. The luciferase activities of PAK5-WT and PAK5-mut reporters in Hep3B and HepG2 cells after transfecting with mimics-NC or miR-138-1-3p mimics measured by dual-luciferase reporter assays. **D** qRT-PCR and Western Blot were conducted to determine the expression of PAK5 in Hep3B-SR and HepG2-SR cells compared with their parental cells. **E** qRT-PCR  and Western Blot were conducted to determine the expression of PAK5 after miR-138-1-3p overexpression or knockdown in Hep3B and HepG2 cells. **F** Western Blot was conducted to determine the expression of PAK5 after the co-transfection as follow in Hep3B and HepG2 cells. **G** and **H** The cell viability and flow cytometric analysis were used to assess the influence after the co-transfection as follow on cell viability and apoptosis in Hep3B and HepG2 cells

To study the detailed molecular mechanisms, TargetScan (http://www.targetscan.org/) and miRTarBase (http://mirtarbase.mbc.nctu.edu.tw/) were used for the prediction of the potential targets. RNA sequencing on HepG2-SR cells and its parental cell lines were also utilized to identify potential targets. Based on the database findings, RNA sequencing and literature review, PAK5 was selected for the further experiment (Fig. 3A). We detected a significant increase of PAK5 in tumor tissue compared with adjacent normal tissues from 40 HCC patients by qRT-PCR (Fig. 3B). Then, a luciferase reporter test was used and we confirmed that PAK5 was an actual target of miR-138-1-3p. We cloned the PAK5 WT 3′-UTR (conserved wild type) or PAK5 mut 3′-UTR (conserved mutant type) into psiCHECK2 vector (psiCHECK2-PAK5 WT 3′-UTR or psiCHECK2-PAK5 mut 3′-UTR) with the upstream luciferase. Significant inhibiting effect was found in the group that was co-transfected with psiCHECK2-PAK5 WT 3′-UTR and miR-138-1-3p mimics in Hep3B and HepG2 cells (Fig. 3C). PAK5 mRNA and protein level were markedly increased in Hep3B and HepG2 sorafenib resistant cells lines validated by qRT-PCR and Western Blot (Fig. 3D). Moreover, miR-138-1-3p mimics inhibited PAK5 expression in Hep3B and HepG2 cells at the protein and mRNA levels. Meanwhile miR-138-1-3p inhibitor promoted PAK5 expression (Fig. 3E). To determine the relation of miR-138-1-3p and PAK5 in HCC sorafenib resistance, we have set three groups of co-transfections, and Western Blot was made for examining the co-transfection effectiveness (Fig. 3F). Cell viability assay was used to analyze their responses to sorafenib. We detected the declining tendency of miR-138-1-3p mimics that was impaired by co-transfection of PAK5 plasmids. Inversely, co-transfected with si-PAK5 eliminated the effect of miR-138-1-3p inhibitor (Fig. 3G). Flow cytometry analyses demonstrated that PAK5 overexpression decreased miR-138-1-3p mimics induced HCC cell apoptosis, and si-PAK5 reduced the effect of miR-138-1-3p inhibitor (Fig. 3H). These data suggested that miR-138-1-3p participated in sorafenib resistance by targeting PAK5.

### PAK5 attenuated cell apoptosis induced by sorafenib in HCC in vitro

**Fig. 4:**
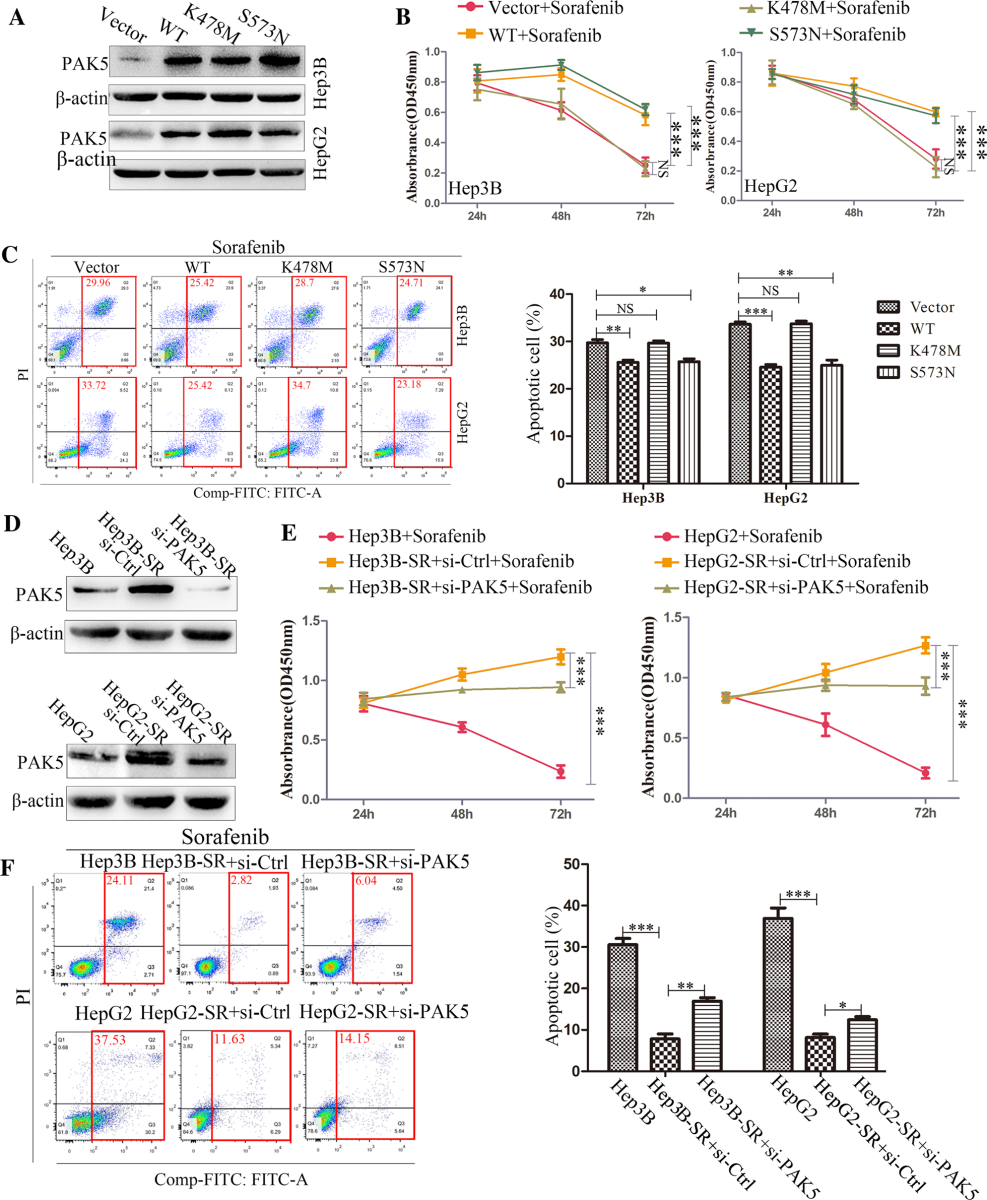
PAK5 attenuated cell apoptosis induced by sorafenib in HCC in vitro. **A** Western Blot was conducted to determine the transfection efficiency of PAK5/Vector, PAK5/WT, PAK5/S573N (constitutively active) and PAK5/K478M plasmids (constitutively inactive) in Hep3B and HepG2 cells. **B** and **C** The cell viability and flow cytometric analysis were used to assess the influence of PAK5 overexpression on cell viability and apoptosis in Hep3B and HepG2 cells. **D** Western Blot was conducted to determine the transfection efficiency of PAK5-siRNA. **E** and **F** The cell viability and flow cytometric analysis were used to assess the influence of PAK5 knockdown on cell viability and apoptosis in Hep3B-SR and HepG2-SR cells

To detect the correlation of PAK5 and HCC sorafenib resistance, we used PAK5/S573N (constitutively active) plasmid and PAK5/K478M (constitutively inactive) plasmid and carried out four groups that were transfected with them, correspondingly, in Hep3B and HepG2 cell lines. Western Blot verified the transfection effectiveness (Fig. 4A). Particularly lower viability of PAK5/K478M-transfected cells was observed in comparison with the PAK5/WT-transfected and PAK5/S573N-transfected cells (Fig. 4B). Sorafenib-triggered apoptosis was also significantly inhibited following the transiently transfection with PAK5/WT or PAK5/S573N (Fig. 4C). These findings indicated that PAK5 mediated the chemotherapy tolerance of sorafenib in HCC.

We also knocked out PAK5 in sorafenib-resistant HCC cells by siRNA, and the interference efficiency was confirmed by Western Blot (Fig. 4D). The cell viability of interfere group was significantly reduced compared with sorafenib-resistance subpopulation, meanwhile higher than their parental cells (Fig. 4E). In addition to that, knockdown of PAK5 stimulated the apoptosis of sorafenib-resistance cells to sorafenib (Fig. 4F). Thus, PAK5 was a critical component in the development of drug-tolerant subpopulation.

### PAK5 promoted ABCB1 transcriptional activation via Wnt/β-catenin signaling pathway

**Fig. 5:**
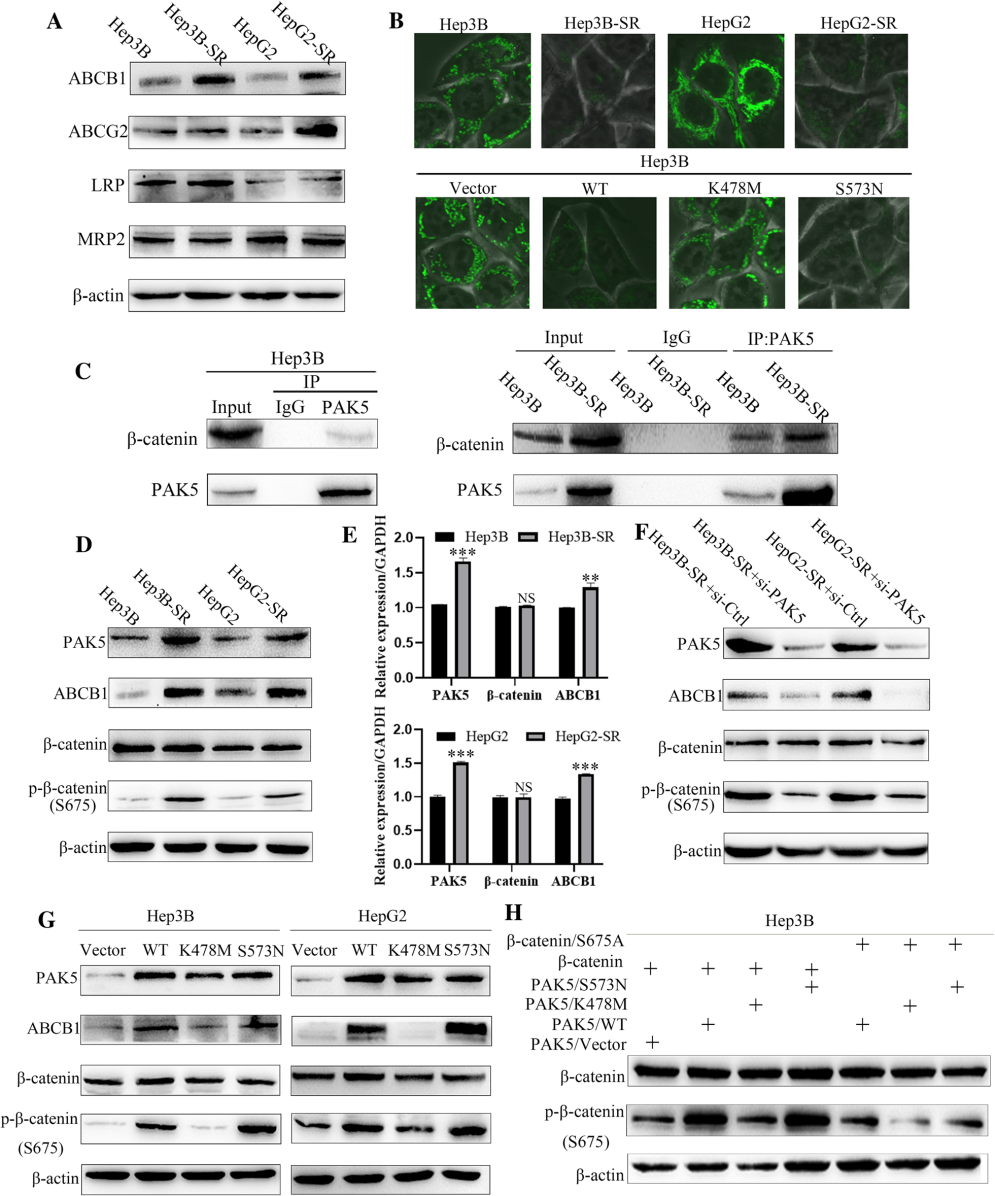
PAK5 promoted ABCB1 transcriptional activation via Wnt/β-catenin signaling pathway. **A** The protein levels of ABCB1, ABCG2, LRP and MRP2 were detected via Western Blot assay in Hep3B-SR and HepG2-SR cells compared with their parental. **B** Fluorescent dye rhodamine 123 (Rho123) as an index to test the P-gp activity in Hep3B-SR and HepG2-SR cells compared with their parental. Fluorescent dye rhodamine 123 (Rho123) as an index to test the P-gp activity in Hep3B cells after transfection with PAK5/Vector, PAK5/WT, PAK5/S573N and PAK5/K478M plasmids. **C** co-IP assay was performed in Hep3B cells by anti-PAK5 antibodies. co-IP assay was performed in Hep3B and Hep3B-SR cells by anti-PAK5 antibodies. **D** The protein levels of PAK5, ABCB1, β-catenin and p-β-catenin were detected via Western Blot assay in Hep3B-SR and HepG2-SR cells compared with their parental. **E** qRT-PCR was conducted to determine the expression of PAK5, ABCB1 and β-catenin in Hep3B-SR and HepG2-SR cells compared with their parental. **F** The protein levels of PAK5, ABCB1, β-catenin and p-β-catenin were detected via Western Blot assay in Hep3B-SR and HepG2-SR cells after PAK5 knockdown. **G** The protein level of PAK5, ABCB1, β-catenin and p-β-catenin was detected via Western Blot assay in Hep3B and HepG2 cells after transfection with PAK5/Vector, PAK5/WT, PAK5/S573N and PAK5/K478M plasmids. **H** The protein levels of β-catenin and p-β-catenin were detected via Western Blot assay in Hep3B cells after the co-transfection of PAK5/Vector, PAK5/WT, PAK5/S573N, PAK5/K478M severally with β-catenin or β-catenin/S675A

We examined several multidrug resistance proteins, including ABCB1, ABCG2, LRP and MRP2, aimed at investigating the expression profiles in Hep3B-SR, HepG2-SR compared with their parental cells. Western Blot revealed a prominently augment of ABCB1 exceeding the other MDR genes in the sorafenib-tolerant subpopulation (Fig. 5A). To determine the functional operation of P-gp in sorafenib-resistance cells, we used fluorescent dye rhodamine 123 (Rho123) as an index to test P-gp activity. Following a period of 30 min, just 10% initial Rh123 fluorescence retained in the intracellular compartment of Hep3B-SR and HepG2-SR, whereas in their parental cells approximately 60%. PAK5/WT-transfected and PAK5/S573N-transfected cells also hold a higher efflux of Rh123 fluorescence when compared with the transfection of PAK5/K478M and PAK5/Vector, which indicated that PAK5 was likely to augment the ABCB1 expression in HCC cell lines (Fig. 5B).

Previous study had shown that PAK5 may promote the vesicle transport in nerve cells by phosphorylate Pacsin1 and Synaptojanin1 [33]. Protein structure prediction of β-catenin had made it clear that there was a PAK5-related identical sequence (K/R) (R/X) X(S/T) in it, and serine 675 constituted a potential PAK5 phosphorylation site [34, 35]. To examine the underlying mechanisms, we performed co-IP assay. Endogenous interaction of PAK5 and β-catenin was detected in Hep3B cells, and more remarkable in sorafenib-tolerant cells (Fig. 5C). Western Blot assay also confirmed it. We transfected the PAK5/Vector, PAK5/WT, PAK5/S573N and PAK5/K478M correspondingly in Hep3B and HepG2 cell lines to the investigation of the protein expression level of ABCB1, β-catenin and p-β-catenin (S675). High expression of ABCB1 and p-β-catenin (S675) was detected in PAK5/WT and PAK5/S573N transfected cells, but the total protein of β-catenin did not alter (Fig. 5D). Also, we observed similar variations in Hep3B-SR and HepG2-SR cells in comparison with their parental cells. A greater extent of p-β-catenin (S675) and a higher expression level of ABCB1 mRNA were detected in the resistant cell lines (Fig. 5E, F). In addition to that, the expression of ABCB1 and p-β-catenin (S675) were attenuated with knocked down PAK5 by siRNA in Hep3B-SR and HepG2-SR cells (Fig. 5G). To test the modulated function of PAK5 on β-catenin signaling pathway, co-transfection of PAK5/Vector, PAK5/WT, PAK5/S573N, PAK5/K478M with β-catenin or β-catenin/S675A (phosphorylation defective mutant) performed on Hep3B cells in accordance with Fig. 5H. These observations verified that PAK5 up-regulated ABCB1 expression via the Wnt/β-catenin signaling pathway.

### PAK5 facilitated the nuclear translocation of β-catenin

**Fig. 6:**
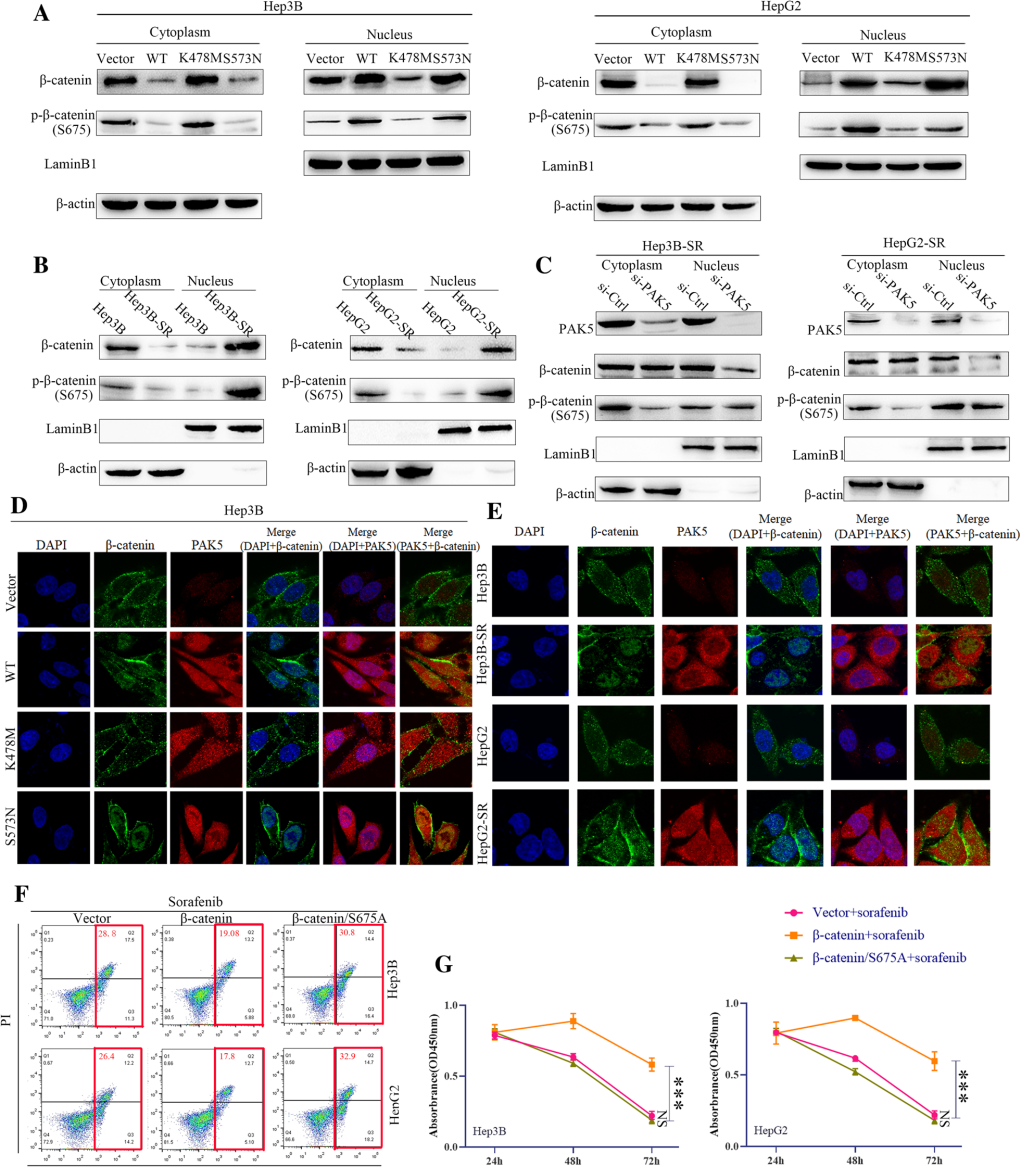
PAK5 facilitated the nuclear translocation of β-catenin. **A** The nuclear and cytoplasmic fractionation were performed to test the cellular distribution of β-catenin and p-β-catenin in Hep3B and HepG2 cells after transfection with PAK5/Vector, PAK5/WT, PAK5/S573N and PAK5/K478M plasmids. **B** The nuclear and cytoplasmic fractionation were performed to test the cellular distribution of β-catenin and p-β-catenin in Hep3B-SR and HepG2-SR cells compared with their parental. **C** The nuclear and cytoplasmic fractionation conducted to test the cellular distribution of β-catenin and p-β-catenin in Hep3B-SR and HepG2-SR cells after PAK5 knockdown. **D** Immunofluorescence was employed to show the cellular distribution of β-catenin in Hep3B cells after transfection with PAK5/Vector, PAK5/WT, PAK5/S573N and PAK5/K478M plasmids. **E** Immunofluorescence was employed to indicate the cellular distribution of β-catenin in Hep3B-SR and HepG2-SR cells compared with their parental. **F** and **G** The cell viability and flow cytometric analysis were used to assess the influence of β-catenin on cell viability and apoptosis in Hep3B and HepG2 cells

We manipulated the nuclear and cytoplasmic fractionation to test the cellular distribution of β-catenin and p-β-catenin (S675) in Hep3B and HepG2 cell lines after transfecting PAK5/Vector, PAK5/WT, PAK5/S573N and PAK5/K478M plasmids, respectively. We found that PAK5/WT and PAK5/S573N significantly facilitated the nuclear distribution of β-catenin and p-β-catenin (S675), and PAK5/K478M transfected cells were similar with PAK5/Vector group (Fig. 6A). We also verified the distribution of β-catenin and p-β-catenin (S675) in Hep3B-SR and HepG2-SR cells (Fig. 6B). Furthermore, silencing PAK5 particularly reduced the phosphorylation of β-catenin and nuclear allocation of p-β-catenin (S675) in Hep3B-SR and HepG2-SR cells. Meanwhile, the dynamical relocation of β-catenin and p-β-catenin was curbed (Fig. 6C). Consistently, the observations of immunofluorescence indicated that the positive signal (red) of PAK5 was strikingly higher in the cytoplasm of PAK5/WT, PAK5/S573N and PAK5/K478M transfected cells. Positive signal (green) of β-catenin predominantly occurred in the nucleus and enhanced in the PAK5/WT and PAK5/S573N transfected cells (Fig. 6D). We also observed that Hep3B-SR and HepG2-SR cells shared similarity with PAK5 overexpression (Fig. 6E). In addition to that, β-catenin/S675A was also used to demonstrate that the phosphorylation states correlate with the sorafenib resistance of the HCC cells (Fig. 6F, 6G). These data demonstrated that PAK5 could elevated the phosphorylation and nuclear translocation of β-catenin.

### β-catenin bonded ABCB1 promoter and activated ABCB1 transcription

**Fig. 7:**
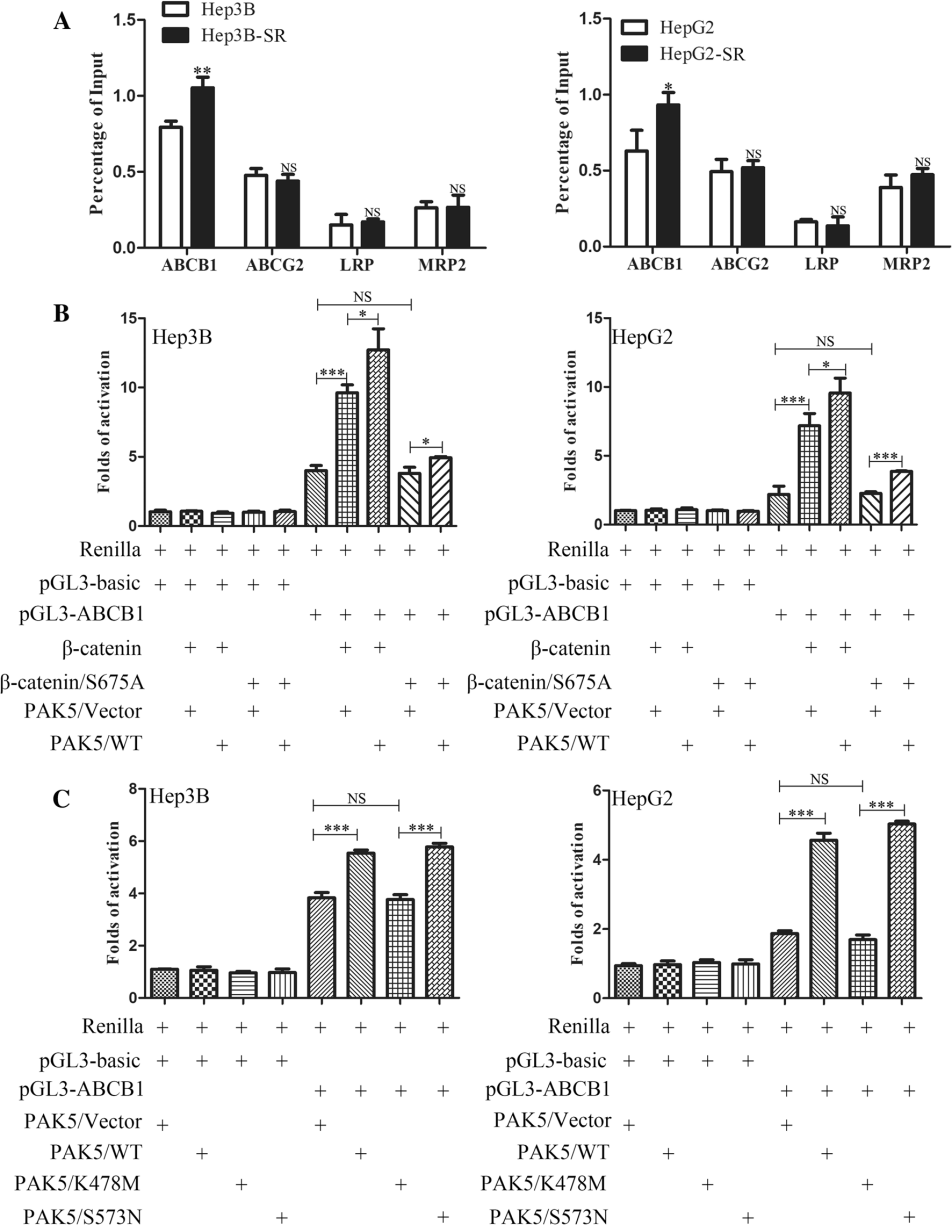
β-catenin bonded ABCB1 promoter and activated ABCB1 transcription. **A** ChIP with β-catenin antibody in Hep3B-SR and HepG2-SR cells and the resulting precipitated DNA samples were quantified by qRT-PCR with ABCB1, ABCG2, LRP and MRP2 primers. **B** and **C** Dual luciferase reporter assay was used to detect the reporter activity after ten or twelve groups’ co-transfected as shown in Hep3B and HepG2 cells

In the canonical Wnt/β-catenin signaling pathway, β-catenin translocated into nuclear to regulate the activity of genes expression. To detect the underlying mechanism of β-catenin in HCC sorafenib resistance, DNA complexes were immunoprecipitated with β-catenin antibody. The precipitated DNA samples were quantified by qRT-PCR with ABCB1, ABCG2, LRP and MRP2 primers. We verified that ABCB1 was a target gene of β-catenin and significantly increased in HCC sorafenib resistance cell lines (Fig. 7A). In addition to that, we demonstrated that β-catenin nuclear translocation promoted ABCB1 promoter activity via dual luciferase reporter assay. (Fig. 7B, C). These findings suggested that PAK5-mediated β-catenin phosphorylation and nuclear translocation increased the transcriptional activity of ABCB1.

### MiR-138-1-3p sensitized sorafenib to HCC by targeting PAK5 in vivo

**Fig. 8:**
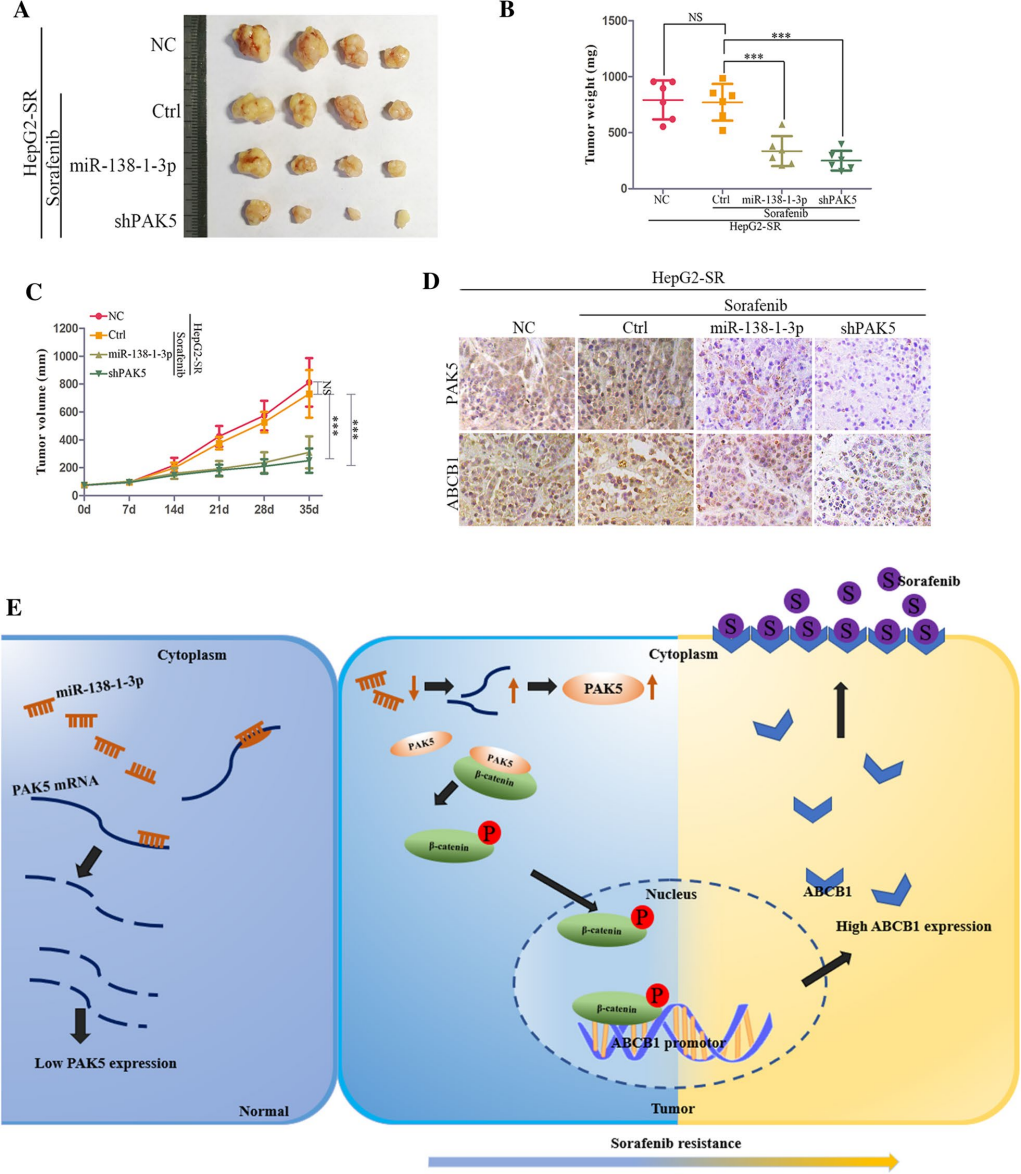
MiR-138-1-3p sensitized sorafenib to HCC by targeting PAK5 in vivo. **A** At 27 days post-inoculation, mice were killed and **B** tumor weights were measured. **C** The tumor volumes were monitored every 7 days. **D** Representative images of PAK5 and ABCB1 immunohistochemical staining with PAK5 and ABCB1 antibodies in four groups as shown. **E** The schematic diagram displayed that MicroRNA-138-1-3p sensitized sorafenib to hepatocellular carcinoma by targeting PAK5 mediated β-catenin/ABCB1 signaling pathway

To evaluate the role of miR-138-1-3p and PAK5 in HCC sorafenib resistance in vivo, xenograft cancer models were established by subcutaneously inoculating HepG2-SR cells. The nude mice were randomly assigned into four groups (n = 10 for each): LV-ctrl-HepG2-SR cells (NC), LV-ctrl-HepG2-SR cells and oral sorafenib (Ctrl), LV-miR-138-1-3p-HepG2-SR cells and oral sorafenib (miR-138-1-3p), LV-shPAK5-HepG2-SR and oral sorafenib (shPAK5). After tumor growth reached a volume of 100 mm^3^, the last three groups’ mice were introduced to oral sorafenib 30 mg/kg, twice a day as well as the first group mice treated with placebo. Compared with miR-138-1-3p overexpressed and shPAK5 groups, the tumor size and weight of NC and Ctrl groups were increased significantly. No statistical difference was observed in LV-ctrl-HepG2-SR groups that treated with sorafenib or not (Fig. 8A–C). Immunohistochemical assay was used in the xenograft tissues that isolated from nude mice. In line with our in vitro experiment, PAK5 and ABCB1 positive signal dampened entirely in miR-138-1-3p overexpressed xenografted tumors. Concordantly, ABCB1 positive signal also subdued in PAK5 inhibited xenografted tumors (Fig. 8D). Thus, we demonstrated that miRNA-138-1-3p sensitizes sorafenib to hepatocellular carcinoma by targeting PAK5 mediated β-catenin/ABCB1 signaling pathway (Fig. 8E).

## Discussion

HCC is one of the leading causes in global cancer deaths. Surgical resection or liver transplantation is considered as the preferred therapy for HCC patients. Nonetheless, majority of the HCC patients had missed the best treatment time due to early onset, late diagnoses, fast-paced progress and high malignancy [36]. Sorafenib is emerging as a pivotal part of HCC treatment, however, the clinical response to sorafenib is largely limited by drug resistance [37]. Our works aimed at examining the cellular alterations and the molecular mechanisms of sorafenib resistance in HCC.

MicroRNAs (miRNA), a class of natural RNA-interfering agents, have been demonstrated associated with all stages of cancers, including tumorigenesis, progression, metastasis and drug resistance [38–41]. And a push for miRNA study in medical science has caused worldwide concern from cancer researchers for the purpose of finding a new therapeutic target [42]. Thus, we identified the low expression miRNAs by RNA sequencing in HepG2-SR cells compared with its parental cell line. We also transfected the resistant HCC cell lines with miRNAs mimics and analyzed their responses to sorafenib respectively by CCK8 assay. MiR-138-1-3p was the specific candidate that not only low expression in sorafenib resistance cells, but also promoting sorafenib induced apoptosis. MiR-138-1-3p is a member of the miR-138 family. Both the miR-138-1 and miR-138-2 clusters responds to the entirely identical mature RNA sequences, but located on the different chromosomes, 3p21.33 and 16q13 correspondingly [43]. The dynamic modulation of miR-138 was detected in multiple disease, like type-2 diabetes, rheumatoid arthritis, early-onset Alzheimer’s disease, and especially cancers [44–48]. Accumulating evidence suggested that miR-138 regulated cancer cells proliferation by inhibiting target genes expression that was implicated in some pro-cancer signaling pathways, including insulin-like growth factor (IGF), EGFR, AKT and MAPK [49–52]. Meanwhile, miR-138 also implicated in modulating the biological functions of cancer cells by targeting several genes expressions. For example, miR-138 leaded to cell cycle arrest by targeting cyclin D3 [37, 53], miR-138 inhibited HCC development via repressing stemness factor SOX9 expression [54], and miR-138 attenuated the adaption of cancer cells to hypoxic microenvironment by affecting the expression of HIF-1α and vascular endothelial growth factor (VEGF) [55, 56]. Moreover, miR-138 inhibited cell migration, invasion and EMT in cancer via directly targeting rhomboid domain-containing protein 1 (RHBDD1) and Mowat-Wilson syndrome-associated transcription factor Zeb2 (Sip1/Zfhx1b), which decreased the E-Cadherin expression and increased the expression of N-Cadherin and Vimentin [57, 58]. Concordantly, all the above biological functions were related with the development of sorafenib resistance. A previous study demonstrated that IGF-1 conferred sorafenib resistance to hepatocellular carcinoma cells by regulating RAS/RAF/ERK signaling pathways [59]. And inhibiting EGFR pathway activity could increase patient sensitivity to sorafenib [60]. Similarly, AKT and MAPK have emerged as critical players in the sorafenib resistance process [61, 62]. Of note, enhancing cancer stem cell properties also promoted sorafenib resistance in HCC [63]. In addition to that, HIF-1α/VEGF pathway adapted HCC cells to hypoxic microenvironment and blunted the antiangiogenic actions of sorafenib [64]. Studies also indicated that sorafenib resistant HCC cells showed EMT characteristics with the downregulation of epithelial marker (N-Cadherin and Vimentin) and upregulation of mesenchymal makers (E-Cadherin) [65]. Stemness, mesenchymal states and hypoxic microenvironment had been identified as microenvironmental and metabolic characteristics of sorafenib resistance in HCC [66]. In the current study, we determined the potential effect of miR-138-1-3p in HCC sorafenib-tolerant cells, and underlined the significance of miR-138-1-3p in liver homeostasis.

Sorafenib functions as a multi-kinase inhibitor, and the development of sorafenib resistance may related with the activity of several kinases [67]. PAK5 was the last identified member of the PAK family and a conserved serine/threonine protein kinase. Based on the database findings, RNA sequencing and literature review, PAK5 was selected for the target of miR-138-1-3p. Several studies have indicated that PAKs involved in diverse kinds of signal pathways that were over-active in cancers [68]. Previous studies confirmed that PAK5 was a key signaling molecule in cancer cells and involved in modulating tumorigenesis, anti-apoptosis and anti-cancer drugs resistance.

HCC is a malignant cancer that lack of specific therapeutic targets now days. However, several pathways were regarded to be important in the development and progression of HCC, including AMPK, AKT, JAK2, mTOR, and β-catenin [69–73]. The differential expression genes in HepG2-SR cells were included for KEGG pathway analysis and eleven pathways were most likely to be targeted. We focused on β-catenin pathway as it was highly involved in HCC development and sorafenib resistance. And protein structure prediction of β-catenin has made it clear that there was a PAK5-related identical sequence (K/R) (R/X) X(S/T) in it, and serine 675 constituted a potential PAK5 phosphorylation site. Then, we verified that PAK5 enhanced the phosphorylation and nuclear translocation of β-catenin that increased the transcriptional activity of multidrug resistance protein ABCB1 in HCC sorafenib resistance cells.

## Conclusions

In summary, our findings demonstrated that miR-138-1-3p negatively regulated PAK5-mediated sorafenib-resistance in HCC cells. Up-regulated PAK5 in sorafenib-resistant subpopulation had a significant correlation with the HCC cell escapism sorafenib lethality. The combination analysis of miR-138-1-3p and PAK5 could be used for the purpose of predicting the sorafenib tolerance, and providing individualized therapy strategies in HCC patients. One limitation of the current study was that sorafenib was mainly used in patients with advanced HCC who usually lost surgery opportunity. Thus, our study was mainly focused on HCC cell lines, which had been already derived in vivo microenvironment and could not be taken into consideration as precise surrogates for medical tumors. Clinical trials are needed before it could be adopted as a treatment.

## Data Availability

All data generated or analyzed during the current study are available from the corresponding author on reasonable request.

## Abbreviations

HCC: Hepatocellular carcinoma
FDA: Food and Drug Administration
MiRNAs: MicroRNAs
PAK5: p21-activated kinases 5
ABC: ATP-binding cassette
qRT-PCR: Quantitative real-time PCR
co-IP: Co-immunoprecipitation
CHIP: Chromatin immunoprecipitation
